# Identifying Preferences for Prostate Cancer Screening Among American Indian Men (Project AIMEPCCo): Protocol for a Discrete Choice Experiment

**DOI:** 10.2196/85095

**Published:** 2026-05-19

**Authors:** Chris Gillette, Ronny Bell, Tony Locklear, Jan Ostermann, Delaney Provenza, Daniel Reuland, Beata Debinski, Kristie Foley, Robert Wooten, Laiton Steele, Quazi Minhaz Tabassum

**Affiliations:** 1 Department of Physician Assistant Studies, Wake Forest University School of Medicine, Medical Center Blvd, Winston-Salem, NC, 27157, United States, 1 3367130820; 2 Division of Pharmaceutical Outcomes and Policy, Eshelman School of Pharmacy, University of North Carolina at Chapel Hill, Chapel Hill, NC, United States; 3 Department of Public Health Education, University of North Carolina at Greensboro, Greensboro, NC, United States; 4 Department of Health Services Policy and Management, Arnold School of Public Health, University of South Carolina, Columbia, SC, United States; 5 Division of General Medicine and Clinical Epidemiology, School of Medicine, University of North Carolina at Chapel Hill, Chapel Hill, NC, United States; 6 Department of Family and Community Medicine, School of Medicine, Wake Forest University, Winston-Salem, NC, United States; 7 Department of Implementation Science, School of Medicine, Wake Forest University, Winston-Salem, NC, United States; 8 Department of Epidemiology and Community Health, University of North Carolina at Charlotte, Charlotte, NC, United States

**Keywords:** American Indian or Alaska Native, prostate cancer, early detection of cancer, shared decision-making, discrete choice experiment, study protocol

## Abstract

**Background:**

American Indian men are disproportionately impacted by prostate cancer (PC) compared to White men and experience the worst PC outcomes of any racial or ethnic group. To address these disparities, it is important to better understand American Indian men’s preferences regarding PC screening.

**Objective:**

The objectives of this study are as follows: (1) conduct a literature review, followed by qualitative, culturally responsive formative research with American Indian men, (2) develop and field a discrete choice experiment (DCE) survey to elicit the preferences of American Indian men toward PC screening, and (3) identify feasible, culturally appropriate, preference-concordant screening strategies that are targeted for American Indian men.

**Methods:**

In this protocol, a scoping literature review to identify previous PC DCEs has been created. We will follow that review by conducting rigorous, theoretically grounded qualitative work to identify plausible DCE attributes and attribute levels among men from the Lumbee Tribe in North Carolina. We will pilot the DCE among Lumbee men to assess cultural responsiveness and comprehension of the choice context and choice tasks. Finally, the DCE methodology will be used to elicit the PC screening preferences among 100 Lumbee men between the ages of 40 and 69 years. Choice data will be analyzed using mixed logit models, and trade-offs will be described using marginal rates of substitution.

**Results:**

The study was funded in 2024. Formative qualitative research is still ongoing. DCE data collection is expected to start in April 2026 and end in June 2026.

**Conclusions:**

This will be the first study to use a culturally responsive approach to DCE development in an indigenous population. The findings of this study can be used to educate providers regarding culturally responsive PC screening and to inform the design of interventions to increase preference-concordant PC screening in American Indian men.

## Introduction

Approximately 10 million people in the United States, or about 3% of the entire population, identify as American Indian or Alaska Native [1]. According to the Centers for Disease Control and Prevention, among American Indian men, prostate cancer (PC) is the second leading cause of cancer-related deaths [2]. Prior research shows that American Indian men are more likely to have advanced distant stage PC at the time of diagnosis compared to non-Hispanic White men. American Indian men have higher PC mortality rates, despite the fact that there is no underlying genetic difference between the 2 racial and cultural identities [3,4]. In North Carolina, recent epidemiological research has found that American Indian men are 72% more likely to die because of PC than non-Hispanic White men [5].

PC is the second most diagnosed cancer among men in the United States and the second leading cause of cancer-related deaths in men [6]. Identifying PC at its earliest stage is associated with a nearly 100% 5-year survival rate [7]. The most common method to screen for PC is through prostate-specific antigen (PSA) testing. PC screening guidelines are heterogeneous and have caused significant controversy around when and whom to screen [8,9]. PSA screening, however, comes with a significant risk of false positives and overdiagnosis (identifying tumors that would not impact a man’s life). Therefore, current guidance from the US Preventive Services Task Force (USPSTF) advises health care providers to use shared decision-making (SDM), starting at the age of 55 years through the age of 69 years, and present the benefits and risks of PSA screening, including overdiagnosis and overtreatment [10]. This is notably different from the 2012 to 2018 USPSTF guidance, which recommended against using PSA-based screening for all men.

While PSA screening has largely fallen out of favor in primary care settings for men with average PC risk, the Prostate Cancer Foundation and the American Urological Association recommend providers to conduct a baseline PSA test for Black men aged between 40 and 45 years and proceed to obtain annual PSA testing if PSA level is at or above 2 ng/mL [11], in contrast to current USPSTF guidelines [12]. The USPSTF guidelines, which are based on the Prostate, Lung, and Colorectal study, do not differentiate between high-risk men and average-risk men, and Black men are not included in their results. So, there is a general misunderstanding about the true sensitivity or specificity of the PSA. Currently, American Indian men are not identified by any organization as a high-risk population for PC, despite experiencing significantly worse outcomes than other racial and ethnic identities.

PSA-based PC screening is considered a preference-sensitive decision because there are multiple medically acceptable options [13]. Preference-sensitive decisions require providers to share the options that patients may have, their risks and benefits, and use SDM to arrive at a decision. Since patient input is required and patients are supposed to share their preferences in these situations, role preferences during medical encounters play an outsized role in these preference-sensitive decisions [14]. However, few studies that investigate patient preferences routinely take into account patients’ role preferences during medical encounters [15].

Persistent disparities in PC mortality due to underscreening, disparities in accessing treatment among American Indian men, and PC diagnoses at later stages of the disease suggest that new approaches are needed to reduce PC mortality in this population. However, less information is known about PC screening preferences, especially in the American Indian population. To understand and address disparities in PC screening rates and outcomes, it is important to study American Indian men’s preferences regarding this important screening test. Discrete choice experiments (DCEs) are routinely used to elicit and quantify preferences for health care decisions, including screening options for cancers and treatments in various real-world scenarios [16,17]. This makes DCE an excellent option to study PC screening preferences.

This study describes our study protocol for a research study aimed at identifying PC screening strategies that align with the preferences of American Indian men and also identifying key tradeoffs. To the authors’ knowledge, this study would be the first to use a culturally responsive approach to develop and field DCE. DCEs offer a novel approach that evaluates how PC screening characteristics interact with individual characteristics and preferences for designing targeted PC screening strategies.

The aims of this protocol report are as follows:

1. Conduct a literature review, followed by qualitative, culturally responsive formative research with American Indian men to develop a comprehensive understanding of facilitators and barriers to PC-related communication, explore role preferences in PC-related SDM, and prioritize preference-relevant features of PC screening strategies;
2. Develop and field a DCE survey to elicit the PC screening preferences of American Indian men and identify barriers to and facilitators of PC screening uptake; and
3. Identify feasible, culturally appropriate, preference-concordant screening strategies targeted for American Indian men.

## Methods

### DCE Overview

The DCE methodology is a rigorous, quantitative, and theoretically grounded approach to elicit preferences. This methodology is based on two theoretical foundations: (1) Lancaster’s consumer theory and (2) random utility theory [18]. The assumption behind Lancaster’s consumer theory is that a person will choose a certain good or service based on an analysis of all its intrinsic characteristics or attributes. The combination of attributes gives rise to utility or value. Random utility theory states that utility (value) is a latent variable that exists but cannot be directly observed. Instead, it is assumed that when an individual chooses a good or service, its utility, that is, the value derived from the combination of all of its attributes, matches or exceeds that of all other alternatives considered. DCEs have been used in numerous pharmacoeconomic, health policy, and consumer preference evaluations in the United States and internationally [19].

The process for developing DCE will follow the guidelines outlined by the International Society for Pharmacoeconomics and Outcomes Research Good Research Practices for Conjoint Analysis Task Force [20].

### Study Design, Participants, and Setting

The study will be conducted in phases, including a systematic literature review, rigorous, theoretically grounded qualitative work, and the design, fielding, and analysis of the DCE. Study participants comprise people of the Lumbee Tribe in southeastern North Carolina. The Lumbee Tribe comprises approximately 55,000 people, making the tribe the largest American Indian tribe in North Carolina and one of the largest tribes in the eastern United States [21]. The Lumbee Tribe has been recently recognized by the federal government but has not yet received federal resources for health care services. Robeson County, where the majority of Lumbee Tribe people live, has the highest prevalence rates of cancers in North Carolina. Previous research has shown that American Indian men statewide in North Carolina have PC incidence rates 72% higher than non-Hispanic White men [5].

### Phase 1: Literature Review

First, our team conducted a scoping literature review to identify previous DCEs in PC screening. The goal of the scoping review was to identify which attributes have been previously included in PC screening DCEs and how attributes were operationalized into levels. We searched for articles in PubMed, Embase, Web of Science, and Global Index Medicus databases. We used the Covidence online web application (Melbourne, Australia) to manage the data. Articles were included in our scoping review if: (1) men or males at birth were included, (2) the outcome used marginal rates of substitution (mRS), latent class analysis, multivariable logit regression, or relative attribute importance, or (3) the study used any of the following types of study designs: (a) conjoint analysis, (b) DCE, or (c) best-worst scale. Our initial search identified 1140 studies, of which 273 were duplicates. Interrater reliability in article selection was Cohen κ=0.88. One author (CG) extracted study findings. *Appendix A* in Multimedia Appendix 1 presents the search strategies that we used to identify the literature. *Appendix B* in Multimedia Appendix 1 presents the data extraction template. Since this was a scoping review, we did not evaluate the quality of the included articles. *Appendix C* in Multimedia Appendix 1 presents the PRISMA (Preferred Reporting Items for Systematic Reviews and Meta-Analyses) flow diagram showing the number of articles identified throughout the search process.

We screened 867 article titles and abstracts and ultimately included 5 studies consisting of 3253 participants in the review (*Appendix C,* Multimedia Appendix 1) [22-26]. There was a median of 6 attributes included in the 5 studies. Table 1 presents the key characteristics of each included study. The most important attribute among all studies was reducing the risk of dying from PC. In the studies that examined an opt-out option (3/5, 60%), which is a realistic choice, most men preferred not to have PC screening in 2 studies [23,26]. In the Charvin et al [23] study, which included an educational video about PSA-based screening, those randomized to the video significantly preferred no screening to getting screened. Howard and colleagues [25] also found that younger men valued preventing PC mortality more than older men, highlighting the need to integrate preference heterogeneity into future studies. Attributes that decreased the likelihood of getting screened for PC included higher risks of needing a biopsy and higher risks of adverse treatment effects. Finally, Pignone and colleagues [26] found that different value clarification methods produced different results on PSA screening decisions; that is, those who received a balance sheet are significantly more likely (*P*<.001) to choose PC screening (43.7%) than a rating and ranking method (34.2%) or DCE (20.2%). However, there was no statistically significant difference between the different methods (balance sheet, rating and ranking, and DCE) on *intention* to undergo PC screening. An important finding of the Pignone study is that when men were presented with unlabeled test options, they most often chose the no screening option. Whereas in the labeled item about whether they intended to get screened for PC, the men chose they intended to get screened. These studies collectively report that most men would prefer not to undergo PC screening given the risks of overdiagnosis and overtreatment. However, the studies also collectively suggest that men are willing to accept tradeoffs such as false positives and overdiagnosis if it means their PC is caught at an early stage.

The most common attributes studied in PC DCEs have been the reduction in PC mortality (N=5) as a result of getting screened, overdiagnosis and overtreatment (4/5, 80%) [22-25], and screening sensitivity (3/5, 60%) [22,25,26]. We adapted the checklist from the Hall et al [16] systematic review of cancer screening DCEs to guide the categorization of attributes (Multimedia Appendix 2). Three out of the 5 (60%) studies examined the adverse effects of PC treatment, which are treatment attributes and not screening attributes [22,25,26]. Only 1 (20%) study included attributes solely focused on PC screening. Crucially, there are no studies that included any attributes regarding the visit or provider that have been shown to be important in clinic visits with American Indian patients, highlighting an important literature gap [27,28]. Future work regarding PC screening in American Indian men needs to include culturally important attributes to ensure cultural responsiveness. Importantly, these studies were conducted in the mid-2010s and there have since been notable improvements in reducing the risks associated with biopsies and the use of magnetic resonance imaging to decide whether a biopsy is even needed [29].

**Table 1.** Key characteristics from prostate cancer discrete choice experiments (total men studied, N=3253).

Study ID	Where conducted	Number of participants	Attributes	Key findings
Berchi and Launoy [22] (2019)	France	1008	Screening technique: PSA^a^ only; PSA with digital rectal examination (DRE)^b^ Mortality reduction: 10% and 20% Capacity to detect cancer: 50%, 70%, and 90% Risk of overdiagnosis due to screening: 20%, 40%, and 60% Risk of impotence due to treatment: 20%, 50%, and 80% Risk of incontinence due to treatment: 5%, 25%, and 40% Cost to the patient (€)^c^: 0, 15, and 30	The most important attribute in increasing the likelihood for prostate cancer screening was mortality reduction. Men had the greatest aversion to PSA only screening compared to DRE and PSA combination screening. Only 4.6% of men chose the no screening option.
Charvin et al [23] (2020)	France	854	Mortality by prostate cancer (per 1000): 2, 5, and 6 False positive result (per 1000): 50, 150, and 250 False negative result (per 1000): 1, 5, and 10 Overdiagnosis (per 1000): 10, 30, and 50 Recommended frequency: every year, every 2 years, and every 4 years Out of pocket costs (€): 0, 10, 20, and 40	Opting out of screening was the most common choice in this sample, chosen by 52.36% of men who watched the informative video. Watching the video increased the likelihood of opting out of prostate cancer screening. Increasing the risk of overdiagnosis increased the probability of choosing the screening option the most. The single most important attribute in the DCE^d^ was reducing prostate cancer mortality.
de Bekker-Grob et al [24] (2013)	The Netherlands	427	Risk reduction of death from PC: 10% relative risk reduction, 20% relative risk reduction, 30% relative risk reduction, and 50% relative risk reduction Screening interval: every year, every 2 years, and every 4 years Risk of unnecessary biopsy: 20%, 40%, 60%, and 80% Risk of unnecessary treatment: 0%, 20%, 50%, and 80% OOP costs (€): 0, 50, 100, and 300	The most important attribute in prostate cancer screening decision was reducing the risk of death from prostate cancer. The latent class model identified 3 latent classes, primarily due to education, willingness to pay for prostate cancer screening, and whether the respondent had anxiety or depression. Men are willing to trade off 2% risk reduction of prostate cancer-related death for 10% lower risk of overtreatment. Those in latent class 3 preferred not getting screened for prostate cancer, whereas those in latent classes 1 and 2 preferred screening compared to no screening.
Howard et al [25] (2015)	Australia and United States	662	Men who will die because of PC: 1, 3, 5/2, 5, 10/20, 30, and 40 Men diagnosed with PC (including overdiagnosed cancers in screened men): 5, 10, 15/100, 150, 200/500, 750, and 1000 Men who have unnecessary prostate biopsies from PSA test false alarms: 10, 20, 30/300, 400, 500/1500, 2000, and 2500 Men who experience ongoing impotence: 820, 835, 850/1350, 1375, 1400/4000, 4150, and 4300 Men who experience ongoing urinary incontinence or moderate/severe bowel problems: 305, 310, 320/580, 600, 650/750, 800, and 850 Approximate OOP cost to you over the next 10 years (AUD)^e^: 0, 1000, and 2500	Preference for avoiding prostate cancer-related death increased the likelihood of screening. Higher age was associated with lower preference for screening compared to no screening. Mortality reduction from prostate cancer was more important for younger men compared to older men. As number of biopsies, incontinence or bowel problems, and costs increased, preference was for no screening.
Pignone et al [26] (2013)	Australia and United States	302	Chance of being diagnosed with prostate cancer over 10 years (per 1000): 40, 60, 80 Chance of dying from prostate cancer over 10 years (per 1000): 2, 3, 4 Chance of having a prostate biopsy as a result of screening over 10 years (per 1000): 0, 240, 330 Chance of becoming impotent or incontinent as a result of screening over 10 years (per 1000): 0, 10, 20	79.8% of respondents in the DCE chose the unlabeled no screening option compared to the unlabeled PSA-style screening option. Intention to have PSA test among those in DCE was still high (73.5%), not statistically different from other survey methods. The most important attribute in the DCE was reducing the likelihood of death from prostate cancer.

a PSA: prostate specific antigen.

b DRE: digital rectal examination.

c The conversion rate in 2026, €1=US $1.17.

d DCE: discrete choice experiment.

e The conversion rate in 2026, Aus $1=US $0.72.

### Phase 2: Formative Qualitative Work to Inform Attribute Selection

#### In-Depth Interviews

Qualitative formative work is considered good practice when designing a DCE [20]. In general, the goal of qualitative research to inform DCE development is to elicit attributes and the potential attribute levels [30]. To better understand the knowledge, beliefs, and attitudes of Lumbee men toward PC screening and to inform the selection of attributes that are relevant to American Indian men, we will conduct in-depth interviews (IDIs). The interview guide (*Appendix D,* Multimedia Appendix 1) based on the Health Belief Model elicits the American Indian men’s perspectives on PC susceptibility, perceptions of the seriousness of PC compared to other health risks, and perceptions on the effectiveness of getting screened for PC. We also used the Two-Eyed Seeing approach to identify culturally relevant aspects and Lumbee men’s attitudes toward sexual health conversations with health care providers and to better understand the context for PC screening decisions. Briefly, the Two-Eyed Seeing approach is an interweaving of indigenous knowledge and settler knowledge, acknowledging that both are important sources of knowledge and can improve health [31]. We will use the IDI findings to identify and prioritize attributes that are important to Lumbee men, with a particular focus on cultural responsiveness and incorporating Lumbee cultural norms.

#### Questionnaire

Participants will also complete a self-administered questionnaire to contextualize the findings from the IDIs. The questionnaire (*Appendix E,* Multimedia Appendix 1) contains items from the HINTS (Health Information National Trends Survey) regarding perceptions of the likelihood of about getting PC and fatalism surrounding cancer diagnoses [29]. Participants will also complete the 1-item control preferences scale to determine role preferences in health care discussions [32]. Participants will then complete a series of items asking them about their preferences for types of health care providers in different scenarios (eg, primary care consultations and sexual health conversations). We will select the scenarios to identify whether there are any cultural norms or practices that would impact Lumbee men’s willingness to engage in conversations about their health and sexual health. Participants will also complete the Wake Forest University Trust in Doctors Short Form, the EuroQol 5D health-related quality of life (EQ-5D-5L), and sociodemographic items [33,34].

#### Participant Sampling and Recruitment

We will complete up to 15 in-person IDIs with Lumbee men in North Carolina. The interviewer is a trusted member of the Lumbee Tribe and a coauthor on this protocol (TL). According to Hollins’ guide, to conduct formative qualitative research for DCE attribute development, the emphasis is not on having a large sample size but to ensure representativeness in experience [30]. Thus, we will include American Indian men who have not been screened for PC, as well as men who have completed screening at least once, and those who have had PC. We obtained permission from the Lumbee Tribal Council prior to obtaining approval from our Institutional Review Board (IRB) and have partnered with the Lumbee Tribe and the local health system to help recruit participants for the IDIs. The IRB of the Wake Forest University School of Medicine is the primary IRB for our research.

#### Data Collection

The IDIs will be audio-recorded and transcribed into text to facilitate analysis. Interviews will be conducted virtually or in-person and audio-recorded.

#### Data Analysis

We will use a rapid reflexive thematic analysis using both inductive and deductive coding to analyze the qualitative data. The theoretical lens through which we will analyze the data includes using the Two-Eyed Seeing approach to ensure cultural responsiveness [31]. The team will use a codebook made of a priori structural codes based on the interview guide. The self-administered survey data will be analyzed using means and SDs for quantitative variables and percentages and frequencies for categorical variables. At the end of phase 2, we will have identified and prioritized key characteristics influencing PC screening among Lumbee men for inclusion in the DCE.

### Phase 3: DCE Development, Piloting, and Fielding

The DCE survey will involve respondents making iterative choices from sets of hypothetical scenarios that are characterized by preference-relevant features identified in phase 2. By systematically varying the attribute levels by means of an experimental design, participants must make tradeoffs between more- and less-preferred characteristics. The patterns of choices are then analyzed to quantify tradeoffs, as well as identify the relative importance of each feature, and predict the likelihood of PC screening uptake.

### Choice Context and Hypothesis

The study aims to identify the key considerations that American Indian men make when deciding about whether to get screened for PC. The choice context presented to the participants in the DCE will be framed as follows:

*Your doctor informs you that there is a blood-based test for PC, and that you are eligible for this test due to your age. Please consider the following scenarios. In which of these scenarios would you be more likely to test for PC?*

We hypothesize that American Indian men evaluate several screening attributes, such as mortality benefit, unnecessary biopsies, and treatment, as well as contextual factors, such as experiences with PC and the manner in which information is presented, when considering PC screening [25].

DCE choice tasks will resemble the task shown in Table 2. The sample task includes attribute levels identified in the literature review and several attribute levels hypothesized a priori to influence the choices of American Indian men; the selection of attributes and attribute levels in the actual DCE will be guided by the findings from phase 2. Columns represent screening options. Rows describe the attributes identified in phase 2. Cells represent feasible levels for each attribute. Each choice task involves the comparison of 2 hypothetical PC screening tests, followed by an “opt-out” question, that is, whether the participant would get tested in the preferred scenario, which reflects a realistic choice based on current USPSTF PC screening recommendations [10]. This opt-out option provides a reference point that allows for estimates of the potential uptake of PC screening. Prior to the choice tasks, participants will be introduced to each attribute, presented with *ceteris paribus* conditions (eg, the participant has insurance that covers the test), and will complete a training and comprehension task.

**Table 2.** Example of discrete choice experiment choice task.

Attribute	Option A	Option B	Option C
Chance of identifying PC^a^ after it has spread	100 out of 1000 men like you would have PC diagnosed after it has spread	200 out of 1000 men like you would have PC diagnosed after it has spread	N/A^b^
Relative risk reduction of PC death from getting screened	Getting screened for PC would reduce the chance that you die because of PC by 25%	Getting screened for PC would reduce the chance that you die because of PC by 10%	Your chance of dying because of PC as a result of not getting screened is about 4%
Risk of false positive	4.6 men like you who get the test would have a biopsy from a false positive test result	2.3 men like you who get the test would have a biopsy from a false positive test result	N/A
Health care provider suggestion	Your doctor asks you whether you want to get screened	Your doctor suggests you get screened	Your doctor asks you whether you want to get screened
Which option do you prefer?	Option A	Option B	Option C

a PC: prostate cancer.

b Not applicable.

### Outcome Measure

#### Primary and Secondary Outcomes

The main survey outcome of interest is the PC screening uptake, and the main analytic outcome is the association between each attribute level and the probability of PC screening uptake. The secondary outcome, which is the tradeoffs that American Indian men are willing to make when deciding about PC screening, will be described in the form of mRS. Results will be used to estimate participants’ relative preferences for all potential combinations of attribute levels, that is, PC screening. Feasible strategies can be ranked in terms of expected PC screening uptake, providing concrete guidance on the acceptability of alternative scenarios, and prioritizing specific strategies to increase PC screening uptake in Lumbee men.

#### Experimental Design and Choice Set Construction

Each respondent will be asked to complete 10 choice tasks. The attributes that will be tested include: (1) chance of dying from PC, (2) screening interval, (3) side effects of PC treatment, (4) risk of false positives, and (5) chance of finding harmful cancer. We will use 2 choice tasks designed to monitor attention and ensure participants understand the choice tasks; the first choice task will include a dominant choice and will be repeated halfway through the set of choice tasks. Robust statistical results can be obtained from a fractional factorial design of several dozen questions. Formally, we will use Ngene software (Sydney, Australia) to identify a *d*-efficient, partial factorial design that has good attribute-level balance to ensure that each respondent will see most or all attribute levels. To incorporate preference heterogeneity into the statistical design, the design will be optimized for mixed multinomial logit analysis, with simulations drawing priors from the estimated parameter distributions, which will be initially based on directional hypotheses and updated using relevant information from the phase 2 data. We will use a panel random parameter logit model to account for the inherent panel nature, with 500 Halton draws and normally distributed random parameters. Due to the anticipated small sample size, we will not implement blocking. We will use SurveyEngine survey software to design and host the final DCE. We will only include participants in the analyses if they answered all of the choice tasks.

#### Pretesting and Pilot Testing

Prior to fielding, the DCE surveys will be pretested and iteratively revised using cognitive interviews. Participants will be asked to “think aloud” while completing the DCE survey; interviewers will evaluate understanding and probe why selected answers were chosen. We will pay particular attention to the framing and cultural responsiveness of the selected attributes and levels during the cognitive interviews. Our research team has substantial experience with the development, deployment, and analysis of DCEs, and the development of this particular DCE survey will benefit from lessons learned in our prior DCEs [35-38]. Two members of the research team (RB, TL) are members of the Lumbee Tribe and conduct cancer prevention research with Lumbee Tribal members.

#### Supplemental Survey

Participants will also complete a survey that assesses potential correlates of preferences. The survey will elicit sociodemographic characteristics (eg, age), health-related quality of life, trust in providers, decisional control preferences, and fatalism (see phase 2).

#### Sample Size and Statistical Power

Based on our past experience and DCE sample size recommendations, which combine the number of respondents (n), choice tasks (t=10), alternatives per task (a=2), and the maximum number of feature levels (c=4), and established a cutoff (nta/c≥500), we calculated that a sample size of 100 participants will provide sufficient power to characterize mean preferences for PC screening [39].

#### DCE Questionnaire Fielding

We will work with the Lumbee Tribe to distribute the online survey and advertise it to Lumbee men. Specifically, we will use a combination of social media, direct emails, flyers, and advertisements at provider offices, and participation in cultural events. Assuming a 25% response rate to the online survey [40], we will distribute the online DCE survey to 400 American Indian men in the Lumbee Tribe in order to reach 100 completed surveys.

#### DCE Data Analysis

We will analyze the choice data using mixed (random parameter) logit models [20]. The binary dependent variable will be 1 for the more preferred alternative and 0 for the other alternative in each choice task. The independent variables will be the effects-coded levels of each attribute. The mixed logit specification, estimated in STATA (StataCorp) version 16 or higher, allows for variation in preferences across respondents as well as error correlation across choice tasks within respondents. If we obtain more than 100 responses, we will also test latent class models to analyze the data, which may provide a better model fit. Latent class models will be estimated using Latent Gold Choice 5.0 software (Statistical Innovations).

The preference weights estimated using mixed logit models are the primary outcome of phase 3. Positive parameter estimates for effects-coded attribute levels indicate that an attribute level is more preferred compared to the mean, while negative parameter estimates indicate aversion. Tradeoffs will be described in the form of mRS.

### Ethical Considerations

This study has been approved by the IRB of Wake Forest University School of Medicine (IRB00084519). We will seek approval from the Lumbee Tribe’s IRB for the DCE phase as well as official approval to start data collection from the Lumbee Tribe. One of the team members of this study is a member of the Lumbee Tribe IRB and will serve as a liaison between the study team and the IRB. The Lumbee Tribe IRB was set up after our study began. All phases of the research study that involve human participants will be conducted in accordance with the Declaration of Helsinki and the Belmont Report. All identities of the participants’ survey and IDI data will be blinded to the research team to ensure anonymity. In phases 2 and 3, Lumbee men will provide informed consent to participate in the study. We will also use Indigenous Data Sovereignty Principles and acknowledge that the Lumbee Tribe is the ultimate authority on how the research is conducted [41]. We also acknowledge that the Lumbee Tribe is an equal partner in this project. All surveys will be confidential and will not contain any identifying information. Regarding the qualitative formative work, all participant references to specific people and places will be replaced with “xx” to ensure anonymity. Data will be transferred through the use of the Health Insurance Portability and Accountability Act–compliant Box cloud software (Box Inc). Participants will receive $50 remuneration for participating in the formative qualitative work. Participants will receive $30 for completing the DCE survey.

## Results

The project was funded in 2024 by the Donaghue Foundation Greater Value Program. The project is anticipated to start data collection in April 2026 and end in June 2026. We have not recruited any participants as of yet into the DCE study. We expect the results to identify preferences of American Indian men that impact PC screening using PSA that would enable future researchers and providers to tailor culturally responsive interventions to increase PC screening in this population. All results from the qualitative formative interviews and the DCE will be shared with the Lumbee Tribe prior to submission to peer-reviewed journals.

## Discussion

### Overall Contribution

This study will be the first to use DCE methods to inform the development of preference-concordant screening strategies aimed at reducing the PC mortality disparity among American Indian men in the United States. We chose to focus on the Lumbee Tribe due to multiple factors. First, American Indians are an underrepresented group in PC research. Second, the Lumbee Tribe is located primarily in rural southeastern North Carolina; rural areas are disproportionately impacted by cancer mortality [5]. Although rural areas in the United States have lower cancer incidence than urban areas, cancer mortality is higher in rural areas [42]. Third, prior work has identified that American Indian men in North Carolina are at higher risk for dying because of PC than other racial and ethnic groups, possibly due to seeking care when their PC is at a more advanced stage. Therefore, new approaches to PC screening are needed. Fourth, this is the first study to tailor a DCE on cancer screening using culturally responsive approaches, and it is being driven by members of the Lumbee Tribe who are cancer prevention researchers (RB, TL). This DCE is the first of its kind and will advance what is known about PC screening beliefs and practices of American Indian men. Estimates from our study will inform provider-patient communication and provider behaviors in PC screening consultations with American Indian men. The findings of this study can be used to design preference-concordant screening strategies and pragmatic trials that compare the effectiveness of different options and incentives to increase PC screening.

### Dissemination of Findings

Prior to submitting the findings of phases 2 and 3, we will inform the Lumbee Tribe of our findings and ask for permission to submit the report to a peer-reviewed journal, in accordance with Indigenous Data Sovereignty Principles [41]. We acknowledge that the Lumbee Tribe has the ultimate authority over where and how research findings that relate to the Lumbee Tribe are disseminated and respect that principle.

Prior DCEs investigating PC screening have found that, among the attributes studied, decreasing one’s chances of dying because of PC is the most important attribute. We hypothesize that the results from this study will be consistent with that principal finding. Our study, however, will provide a culturally responsive approach to PC screening among American Indian men. The information from our study will inform future interventions with providers and American Indian men to increase PC screening.

The strength of our study is that it is being developed in collaboration with and under the approval of the Lumbee Tribe of North Carolina, one of the largest tribes in the eastern United States. The area in which members of the Lumbee Tribe reside is also rural, providing one of the first estimates of, which we are aware of, PC screening preferences in this population.

Like all studies, our DCE has limitations. First, indigenous tribes are incredibly diverse, and the findings from one tribe are not expected to apply to other tribes. Second, the low expected sample size precludes any possibility of investigating preference heterogeneity and latent class analysis. Additionally, while our attributes and approach have been guided by formative qualitative interviews, there may be other aspects that inform preferences of American Indian men in PC screening that were not captured and thus excluded from our DCE.

## Supplementary material

10.2196/85095 Multimedia Appendix 1 Literature search strategies and interview guides.

10.2196/85095 Multimedia Appendix 2 Attributes used in prior prostate cancer discrete choice experiment.

10.2196/85095 Peer Review Report 1 Peer-review report from the Greater Value Portfolio Grant Committee, Patrick and Catherine Weldon Donaghue Medical Research Foundation (The Donaghue Foundation (USA).

## Abbreviations

DCE: discrete choice experiment
HINTS: Health Information National Trends Survey
IDI: in-depth interviews
IRB: Institutional Review Board
mRS: marginal rates of substitution
PC: prostate cancer
PRISMA: Preferred Reporting Items for Systematic Reviews and Meta-Analyses
PSA: prostate specific antigen
SDM: shared decision-making
USPSTF: US Preventive Services Task Force
