# Supplementary material for: Identifying Preferences for Prostate Cancer Screening Among American Indian Men (Project AIMEPCCo): Protocol for a Discrete Choice Experiment
Source: JMIR Res Protoc. 2026 May 19;15:e85095. doi: 10.2196/85095 (PMC13186530; doi:10.2196/85095)
Supplement: Multimedia Appendix 1 [file resprot-v15-e85095-s001.docx]

***Appendix A:*  *Prostate Cancer Screening Discrete Choice Experiment Systematic Review Search Strategies***

| **Database** | **Search Strategy** |
| --- | --- |
| Pubmed/Medline | (("Prostatic neoplasms"[Mesh] OR "prostate cancer"[tiab] OR "cancer of the prostate"[tiab] OR "prostate tumor"[tiab] OR "prostatic tumor"[tiab] OR "prostatic adenocarcinoma"[tiab] OR "prostate carcinoma"[tiab] OR "prostatic carcinoma"[tiab] OR "cancerous prostate"[tiab] OR "cancers of the prostate"[tiab] OR "prostatic cancer"[tiab] OR "prostatic neoplasms"[tiab]) AND ("Early detection of cancer"[Mesh] OR screen*[tiab] OR "cancer prevention"[tiab] OR "cancer detection"[tiab] OR "detection"[tiab])) AND ("discrete choice experiment"[tiab] OR "conjoint analysis"[tiab] OR "stated preference"[tiab] OR "choice experiment"[tiab] OR "attribute"[tiab] OR "choice method"[tiab] OR "preference"[tiab]) |
| Embase | ('prostate tumor'/exp OR 'prostate cancer':ti,ab,kw OR 'cancer of the prostate':ti,ab,kw OR 'prostate tumor':ti,ab,kw OR 'prostatic tumor':ti,ab,kw OR 'prostatic adenocarcinoma':ti,ab,kw OR 'prostate carcinoma':ti,ab,kw OR 'prostatic carcinoma':ti,ab,kw OR 'cancerous prostate':ti,ab,kw OR 'cancers of the prostate':ti,ab,kw OR 'prostatic cancer':ti,ab,kw OR 'prostatic neoplasms':ti,ab,kw) AND ('early cancer diagnosis'/exp OR 'screen*':ti,ab,kw OR 'cancer prevention':ti,ab,kw OR 'cancer detection':ti,ab,kw OR 'detection':ti,ab,kw) AND ('discrete choice experiment':ti,ab,kw OR 'conjoint analysis':ti,ab,kw OR 'stated preference':ti,ab,kw OR 'choice experiment':ti,ab,kw OR 'attribute':ti,ab,kw OR 'choice method':ti,ab,kw OR 'preference':ti,ab,kw) |
| Web of Science | TS=("Prostatic neoplasms" OR "prostate cancer" OR "cancer of the prostate" OR "prostate tumor" OR "prostatic tumor" OR "prostatic adenocarcinoma" OR "prostate carcinoma" OR "prostatic carcinoma" OR "cancerous prostate" OR "cancers of the prostate" OR "prostatic cancer" OR "prostatic neoplasms") AND TS=("Early detection of cancer" OR screen* OR "cancer prevention" OR "cancer detection" OR detection) AND TS=("discrete choice experiment" OR "conjoint analysis" OR "stated preference" OR "choice experiment" OR attribute OR "choice method" OR preference) |

***Appendix B: Scoping Review Extraction Template***

| **Covidence ID** |
| --- |
| **Study ID** |
| **Title** |
| **Country in which study was conducted** |
| **Notes** |
| **Type of study** |
| **Aim of study** |
| **Study Funding Sources** |
| **Population** |
| **Inclusion criteria** |
| **Exclusion criteria** |
| **Total number of men studied** |
| **Attributes Studied (1-10)** |
| **Attribute Levels (1-7)** |
| **Number of attributes** |
| **Number of choice tasks** |
| **Major findings** |

***Appendix C: Prostate Cancer DCE PRISMA Flow Chart***

Studies from databases/registers **(n = 1140)**

Web of Science (n = 712)

Embase (n = 269)

PubMed (n = 159)

**Identification**

Studies included in review **(n = 5)**

Studies excluded **(n = 846)**

Studies not retrieved **(n = 0)**

Studies assessed for eligibility **(n = 21)**

Studies sought for retrieval **(n = 21)**

Studies screened **(n = 867)**

Studies excluded **(n = 16)**

Wrong outcomes (n = 6)

Wrong study design (n = 10)

References removed **(n = 273)**

Duplicates identified manually (n = 5)

Duplicates identified by Covidence (n = 268)

Marked as ineligible by automation tools (n = 0)

Other reasons (n = )

**Screening**

**Included**

***Appendix D: Patient Interview***

**AIMEPCCo: American Indian Men and Prostate Cancer Communication:**

**FORM C: Patient Interview Guide**

**INTERVIEW INTRODUCTION**

I want to start by thanking you for helping us with this project today. I will be asking questions about your experiences with prostate cancer screening and your opinions on reasons why men do and do not get screened. We know not everyone who is eligible to be screened has done so. It is important to us to hear from men who have been screened and those who have not. I am not here to judge any decisions you have made, or why you made those decisions. We are also trying to understand experiences of American Indian men more broadly and how to improve healthcare for American Indian men and specifically Lumbee men in this area.

The interview will take about 45-60 minutes. There are no right or wrong answers. We want to learn from you to be able to explore how to make the process of prostate cancer screening better for patients. I would like to be able to audio-record our conversation; this is so that I or one of my colleagues can go back to listen to make sure we heard everything you say.

I want to remind you that you can skip any questions you do not want to answer and stop the interview at any time. All of your responses with be anonymous; your name will not be linked with any of your responses. This includes both when we save the audio-recording, and if we write about or present what you and other men share.. The recording and notes will be kept confidential.

Do you have any questions?

***[Begin Recorder]*** This is participant ID *[insert ID number here] for* American Indian Men and Prostate Cancer Communication. Do I have your permission to record this interview? *[pause]* I want to confirm that we reviewed the consent information, you have received a copy of the form, and you gave your consent to participate. *[pause]*

The interview will first ask you some Yes/No questions and then, based on your responses, ask a series of open-ended questions. Are you ready to get started?

The recommended test for prostate cancer screening is a blood test, called a prostate-specific antigen test, or PSA test. Sometimes, providers may also do a digital rectal exam also called a “DRE.” A digital rectal exam is where a health care provider inserts a lubricated, gloved finger into a man’s rectum to feel the prostate for lumps. For this interview, we will mostly focus on the PSA test for prostate cancer screening.

Have you ever heard of a PSA test or prostate cancer screening before this interview? (y/n)

- 1. If NO, go to Question Set A

Have you ever had a conversation with a primary care provider about prostate cancer screening? (y/n)

- 1. If NO, go to Question Set A

Have you ever, to your knowledge, been screened for prostate cancer using a PSA? (y/n)

- 1. If NO, go to Question Set B
  2. If YES, go to Question Set C

**Question Set A (No Prior Knowledge, No Prior PCP Discussion)**

I would like to start with some general questions related to prostate cancer.

1. Has anyone you know had a prostate cancer test or been diagnosed with prostate cancer?
   1. Is prostate cancer something that you or someone you know would discuss amongst friends or family?
2. How would you compare your risk of prostate cancer to other risks to your health? These could include heart disease, lung cancer, high cholesterol, or other health issues?
   1. *Possible probe:* Why do you think the risk is higher/lower?
3. When you think about prostate cancer, what impact do you think it has on someone’s life, especially for American Indian men?
4. Is there anything else you would like to discuss about what you’ve heard or know about prostate cancer?

Next, I would like to ask some questions about your preferences for healthcare.

1. Do you have a person whom you think of as your personal health care provider/doctor, or a primary care provider (PCP)? (y/n)
2. What do you look for in a doctor or a health care provider?
   1. *Possible probes*: Does it matter to you if they are the same gender as you? About the same age as you? Does it matter if they are American Indian?
   2. *If indicate any factors matter, can probe:* Why is this (are these) important?
3. If HAVE PCP: How comfortable are you talking with your PCP about prostate cancer screening?
   1. *Can probe:* Why/why not?
4. If NO PCP, would you feel comfortable talking about prostate cancer screening with a provider you have not met?
   1. *Can probe:* Why/why not?
5. Do you find it difficult to talk about sexual health problems with a doctor/healthcare provider?
   1. *Probe:* What makes it difficult to talk about sexual health?
   2. *Probe:* What would make it easier to talk with a provider about sexual health?
6. What information would you need, to feel you would be ready to have a conversation about cancer with your doctor/provider?
   1. What about for getting a prostate cancer screening test?
7. Is there anything else you would like to discuss about what is important to you in your interactions with providers or doctors? This could be related to prostate cancer or sexual health, or not.

The last set of questions for this part of the interview are about what you think matters about healthcare providers to other men, and especially to other American Indian or Lumbee men?

1. Do you think provider characteristics would matter to other men in deciding whether to talk about prostate cancer screening with a provider?
   1. If YES, which ones? Which makes the most difference?
2. Are there cultural issues that make it difficult for American Indian men to talk about sexual health, or prostate cancer with their doctors? Or to talk about getting screened for prostate cancer?
   1. *Possible probe:* Are there any issues with talking about [erectile dysfunction, rectal exam, side effects of treatment]?
3. Is there anything else you think is important for us to know about how American Indian men engage with prostate cancer screening?

Is there anything we haven’t asked about today that we should have? Is there anything else you would like to discuss?

**Question Set B (Prior PCP Discussion, No Screening)**

I would like to start with some general questions related to prostate cancer.

1. Has anyone you know had a prostate cancer test or been diagnosed with prostate cancer?
   1. Is prostate cancer something that you or someone you know would discuss amongst friends or family?
2. How would you compare your risk of prostate cancer compared to other risks to your health? These could include heart disease, lung cancer, high cholesterol, or other health issues.
   1. Possible probe: Why do you think the risk is higher/lower?
3. When you think about prostate cancer, what impact do you think it has on someone’s life, especially for American Indian men?
4. Is there anything else you would like to discuss about what you’ve heard or know about prostate cancer?

Next, I would like to ask you some questions about your previous experience getting a PSA (prostate-specific antigen) test.

1. Do you have a person whom you think of as your personal care provider/doctor, or a primary care provider (PCP)? (y/n)
2. Please think back to the discussion you had about prostate cancer screening. Was the conversation that you had about prostate cancer screening with someone who you think of as your personal health care provider/doctor (i.e. primary care provider (PCP)? (y/n)
   1. If NO, with whom?
   2. If YES, did you feel comfortable talking about prostate cancer screening with them?
      1. Why/why not? *[prompt on whether the provider’s gender, racial identification, etc. influenced decision for or against talking with them about it]*
   3. What can you remember about what the provider told you about the PSA or prostate cancer screening in general? [potential prompts include]
      1. Did the provider tell you that you could choose whether or not to have a PSA? Or that some doctors recommend the PSA and other doctors do not recommend men get screened?
      2. Did the doctor tell you that no one is sure if using the PSA actually saves lives?
      3. Did your doctor ask you whether or not you wanted to have a PSA test?
      4. What do you remember about what the doctor/provider said about the accuracy of the PSA test? What do you remember about what the doctor/provider said about different types of prostate cancer?
      5. Did your doctor tell you that actually treating any type of prostate cancer can lead to serious side effects, such as problem with having sex or urination?
3. What was the reason or reasons why you chose not to get a PSA?
   1. Did anything about the provider influence your decision?
4. Is there any information or circumstance that would change your decision and get a prostate cancer screening test?
5. Is there anything else you would like us to know about your experience in discussing prostate cancer screening with a provider?

The last set of questions for this part of the interview are about what you think matters about healthcare providers to other men, and especially to other American Indian or Lumbee men.

1. Do you think provider characteristics would matter to other American Indian men in deciding whether to talk about prostate cancer screening with a provider?
   1. If YES, which ones? Which makes the most difference?
2. Are there cultural issues that make it difficult for American Indian men to talk about sexual health, or prostate cancer with their doctors? Or about getting screened for prostate cancer?
   1. *Possible probe:* Are there any issues with talking about [erectile dysfunction, rectal exam, side effects of treatment]?
3. What are some reasons you believe other men, and specifically American Indian men, may choose not to get a PSA?
   1. *Probe:* Tell me more, or why do you think that?
   2. *Probe:* What specifically about the PSA blood test do you think might matter the most in the decision to get or not get the PSA?
4. Is there anything else you think is important for us to know about how American Indian men engage with prostate cancer screening?

Is there anything we haven’t asked about today that we should have? Is there anything else you would like to discuss?

**Question Set C (History of Screening)**

1. Has anyone you know had a prostate cancer test or been diagnosed with prostate cancer?
   1. Is prostate cancer something that you or someone you know would discuss amongst friends or family?
2. How would you compare your risk of prostate cancer compared to other risks to your health? These could include heart disease, lung cancer, high cholesterol, or other health issues.
   1. *Probe:* Why do you think the risk is higher/lower?
3. When you think about prostate cancer, what impact do you think it has on someone’s life, especially for American Indian men?
4. Is there anything else you would like to discuss about what you’ve heard or know about prostate cancer?

Next, I would like to ask you some questions about your previous experience getting a PSA (prostate-specific antigen) test.

1. Do you have a person whom you think of as your personal care provider/doctor, or a primary care provider (PCP)? (y/n)
2. Have you had more than one PSA test?
   1. How long ago was your first test?
   2. How long ago was your most recent PSA test?
3. Please think back to any discussion or discussions you had about prostate cancer screening. Was the conversation that you had about prostate cancer screening with someone who you think of as your personal health care provider/doctor (i.e., PCP)? (y/n)
   1. If NO, with whom?
   2. If YES, did you feel comfortable talking about prostate cancer screening with them?
      1. Why/why not? *Probe on whether the provider’s gender, racial identification, etc. influenced decision for or against talking with them about it OR*
      2. IF NO, what would have made it more comfortable?
   3. What can you remember about what the provider told you about the PSA or prostate cancer screening in general? [potential prompts include]
      1. Did the provider tell you that you could choose whether or not to have a PSA? Or that some doctors recommend the PSA and other doctors do not recommend men get screened?
      2. Did the doctor tell you that no one is sure if using the PSA actually saves lives?
      3. Did your doctor ask you whether or not you wanted to have a PSA test?
      4. What do you remember about what the doctor/provider said about the accuracy of the PSA test? What do you remember about what the doctor/provider said about different types of prostate cancer?
      5. Did your doctor tell you that actually treating any type of prostate cancer can lead to serious side effects, such as problem with having sex or urination?
4. What was the reason or reasons why you chose to get a PSA?
   1. Did anything about the provider affect your decision? (If YES, what/how?)
5. Would you recommend to another man to get screened for prostate cancer?
   1. Why/why not?
6. What do you remember about your previous PSAs and how the doctor communicated the results to you?
   1. Probe: If had a positive test, ask about clinical care that happened afterwards (e.g., specialist visit, biopsy)
7. Is there anything else you would like us to know about your decision to get a PSA test?

The last set of questions for this part of the interview are about what you think matters about healthcare providers to other men, and especially other American Indian or Lumbee men.

1. Do you think provider characteristics would matter to other American Indian men in deciding whether to talk about prostate cancer screening with their provider, or in deciding whether or not to get screened?
   1. If YES, which ones? Which makes the most difference?
   2. IF YES, what would make it easier to talk about prostate cancer screening?
2. Are there cultural issues that make it difficult for American Indian men to talk about sexual health or prostate cancer with their doctors? Or about getting screened for prostate cancer?
   1. *Possible probe:* Are there any issues with talking about [erectile dysfunction, rectal exam, side effects of treatment]?
3. Is there anything else you think is important for us to know about how American Indian men engage with prostate cancer screening?

Is there anything we haven’t asked about today that we should have? Is there anything else you would like to discuss?

***Appendix E: Patient Questionnaire***

**American Indian Men and Prostate Cancer Preferences**

Thank you for your willingness to complete this survey. Please complete the following items. There are no right or wrong answers.

Many of the questions ask about your experience and perspectives on doctors or healthcare providers. Here, healthcare providers can refer to different people you might interact with closely as part of your care and decision making. This can include physician assistants (PA) and nurse practitioners (NP).

Please answer the following items about your prior experiences of prostate cancer discussions with your healthcare provider.

**The first items ask you about your perception of whether or not you will get prostate cancer in the future or any type of cancer in the future.**

How likely do you think it is that you will develop prostate cancer in the future?

Very low

Somewhat low

Moderate

Somewhat high

Very high

Compared to the average man your age, would you say that you are more likely to get prostate cancer, or less likely, or about as likely?

More likely to get prostate cancer

Less likely

About as likely

How often do you worry about getting prostate cancer?

Rarely or never

Sometimes

Often

All of the time

These next few items are asking you about your perceptions regarding getting cancer (any type, not just prostate cancer) in your lifetime. Remember, there are no right or wrong answers.

How likely are you to get cancer in your lifetime?

Very unlikely

Unlikely

Neutral

Likely

Very likely

**The following items are about your preferences for communicating health information. Please select one response.**

In general how easy or hard do you find it to understand medical statistics?

Very easy

Easy

Hard

Very hard

How much do you agree or disagree with the following statements?

In general, I feel uncomfortable with health information that has a lot of numbers and statistics

Strongly Agree

Somewhat Agree

Somewhat Disagree

Strongly Disagree

People can talk about the chance of something happening using either words, like "It rarely happens" or numbers, like "There's a 5 percent chance". When people tell you the chance of something happening do you prefer they use words or numbers?

Prefer Words

Prefer Numbers

No preference

Out of the statements below, which statement most closely aligns with your preferences about making treatment decisions?

I prefer to make the final treatment selection

I prefer to make the final treatment selection after seriously considering my doctor's opinion

I prefer that my doctor and I share responsibility for deciding which treatment is best

I prefer my doctor to make the final treatment decision, but only after my doctor has seriously considered my opinion

I prefer to leave all treatment decisions to my doctor

How much do you agree or disagree with the following statements?

Please answer the following items about your trust in doctors/healthcare providers, in general.

**How much do you agree or disagree with the following statements?**

Strongly Disagree

Disagree

Agree

Strongly Agree

It seems like everything causes

cancer

There's not much you can do to

lower your chances of getting

cancer

There are so many different recommendations about preventing cancer, it's hard to know which ones to follow

When I think of cancer, I automatically think of death

I go to medical appointments expecting the worst

Please answer the following item asking you about your preferences when you and your doctor/healthcare provider are making decisions.

These next items are about your preferences regarding your healthcare providers. Remember, there are no right or wrong answers.

If you had to choose, for your general health, would you prefer to be treated by a healthcare provider of your own gender, or not?

I prefer same gender provider

I prefer different-gender provider

I have no preference regarding gender of provider

If you had to choose, for your general health, would you prefer to be treated by a healthcare provider of your own race or ethnic group, or not?

I prefer same race/ethnicity provider

I prefer different race/ethnicity provider

I have no preference regarding race/ethnicity of provider

If you had to choose, for mens' health issues (e.g., prostate cancer, erectile dysfunction, urinary incontinence), would you prefer to be treated by a healthcare provider of your own gender, or not?

I prefer same gender provider

I prefer different-gender provider

I have no preference regarding gender of provider

If you had to choose, for mens' health issues (e.g., prostate cancer, erectile dysfunction, urinary incontinence) would you prefer to be treated by a healthcare provider of your own race/ethnicity, or not?

I prefer same race/ethnicity provider

I prefer different race/ethnicity provider

I have no preference regarding race/ethnicity of provider

**Please answer the following items about your trust in doctors/healthcare providers or in the healthcare system, in general.**

Strongly Agree Agree Neutral Disagree Strongly

Disagree

Sometimes doctors care more about what is convenient for them than about their patients' medical needs

Doctors are extremely thorough and careful

You completely trust doctors' decisions about which medical treatments are best

A doctor would never mislead you on anything

All in all, you trust doctors completely

**Fore each heading below, please click the ONE box that best describes your health TODAY.**

Please click the ONE box that best describes your health TODAY.

© EuroQol Research Foundation. EQ-5D™ is a trade mark of the EuroQol Research Foundation

Please click the ONE box that best describes your health TODAY.

© EuroQol Research Foundation. EQ-5D™ is a trade mark of the EuroQol Research Foundation

Please click the ONE box that best describes your health TODAY.

© EuroQol Research Foundation. EQ-5D™ is a trade mark of the EuroQol Research Foundation

Please click the ONE box that best describes your health TODAY.

© EuroQol Research Foundation. EQ-5D™ is a trade mark of the EuroQol Research Foundation

Please click the ONE box that best describes your health TODAY.

© EuroQol Research Foundation. EQ-5D™ is a trade mark of the EuroQol Research Foundation

MOBILITY

______

SELF-CARE

______

USUAL ACTIVITIES

______

PAIN / DISCOMFORT

______

ANXIETY / DEPRESSION ______

© EuroQol Research Foundation. EQ-5D™ is a trade mark of the EuroQol Research Foundation

We would like to know how good or bad your health is TODAY.

This scale is numbered from 0 to 100.

100 means the best health you can imagine. 0 means the worst health you can imagine.

Please click on the scale to indicate how your health is TODAY.

0 - The worst 100 - The best © EuroQol Research Foundation. EQ-5D™ is a trade health you can health you can mark of the EuroQol Research Foundation imagine 50 imagine

*(*

*Place a mark on the scale above)*

© EuroQol Research Foundation. EQ-5D™ is a trade mark of the EuroQol Research Foundation

**Has a doctor ever told you that had any of the following medical conditions?**

Yes

No

Heart attack

Congestive heart failure

Problems caused by stroke

Chronic lung disease, asthma,

emphysema, or chronic

bronchitis

Ulcers

Leg pain when walking due to

poor circulation

High blood pressure or

hypertension

Depression or anxiety disorder

Other cancer (within last 5

years)

Disease of the nervous system

(

Parkinson's disease or multiple

sclerosis (MS))

Arthritis

HIV/AIDS

What is your current age in years?

__________________________________

What is the highest grade or level of schooling you completed?

Less than high school

High school graduate

Some college

College graduate or more

Prefer not to say

What is your marital status?

Married

Living with a partner

Divorced

Widower

Separated

Never been married

Which of the following best describe your current occupational status? Mark all that apply.

Employed

Unemployed for 1 year or more

Unemployed for less than 1 year

Home-maker

Student

Retired

Disabled

Other-Please specify ______

Please identify the type of health insurance you currently have (select all that apply):

Insurance through a current or former employer or union (of you or another family member)

Insurance purchased directly from an insurance company (by you or another family member)

Medicare

Medicaid, Medical Assistance, or any kind of government-assistance plan for those with low incomes or a

disability

TRICARE or other military health care

VA (including those who have ever used or enrolled for VA health care)

Indian Health Service

No insurance/self-pay

What is your household's combined annual income, meaning the total pre-tax income from all sources earned in the past year?

Less than $20,000

$20,000 to < $35,000

$35,000 to < $50,000

$50,000 to < $75,000

$75,000 or more

Prefer not to say

Please identify your ethnic identity

Hispanic/Latino

Non-Hispanic/Latino
