# Supplementary material for: Identifying Preferences for Prostate Cancer Screening Among American Indian Men (Project AIMEPCCo): Protocol for a Discrete Choice Experiment
Source: JMIR Res Protoc. 2026 May 19;15:e85095. doi: 10.2196/85095 (PMC13186530; doi:10.2196/85095)
Supplement: Multimedia Appendix 2 [file resprot-v15-e85095-s002.docx]

**Table 2: Attributes Used in Prior Prostate Cancer Discrete Choice Experiments**

|  | **Test specific** | | | | | | | | | **Outcomes** | | | | | **Service delivery** | | | | | | | | | | | | **Monetary** |
| --- | --- | --- | --- | --- | --- | --- | --- | --- | --- | --- | --- | --- | --- | --- | --- | --- | --- | --- | --- | --- | --- | --- | --- | --- | --- | --- | --- |
| **Study ID** | **Procedure** | **Preparation** | **Pain/discomfort** | **Recovery time** | **Test reputation** | **Test duration** | **Sensitivity** | **Specificity** | **Scope of test** | **Follow-up testing** | **Mortality risk** | **Overtreatment/diagnosis** | **False positive/false negative** | **Side effects** | **Screening interval** | **Test location** | **Method of invitation** | **Results delivery** | **Pre-test support/info** | **Appointment booking** | **HCP Characteristic** | **Waiting time** | **Travel time** | **Type of HCP** | **Time since last test** | **Other** | **Cost** |
| **Berchi 2020** | x |  |  |  |  |  | x |  |  |  | x | x |  |  |  |  |  |  |  |  |  |  |  |  |  | ✓ | x |
| **Charvin 2020** |  |  |  |  |  |  |  |  |  |  | x | x | ✓ |  | x |  |  |  |  |  |  |  |  |  |  |  | x |
| **De Bekker-Grob 2013** |  |  |  |  |  |  |  | x |  |  | x | x | x |  | x |  |  |  |  |  |  |  |  |  |  |  | x |
| **Howard 2015*** |  |  |  |  |  |  | x |  |  | x | x | x | x |  |  |  |  |  |  |  |  |  |  |  |  | ✓ | x |
| **Pignone 2013** |  |  |  |  |  |  | x |  |  | x | x |  |  |  |  |  |  |  |  |  |  |  |  |  |  | x |  |

| ✓ | Included in multiple attributes |
| --- | --- |
| **x** | Included in 1 attribute |
