# Supplementary material for: Identifying Preferences for Prostate Cancer Screening Among American Indian Men (Project AIMEPCCo): Protocol for a Discrete Choice Experiment
Source: JMIR Res Protoc. 2026 May 19;15:e85095. doi: 10.2196/85095 (PMC13186530; doi:10.2196/85095)
Supplement: Peer Review Report 1 [file resprot-v15-e85095-s003.pdf]

Statistician Feedback:

Evaluation Criteria:
1) Objectives- What are the proposed or desired outcomes of this research study?
2) Methodology- Are the research methods consistent and most appropriate for the proposed research?
3) Validity- Can the results of the proposed research be generalizable?
4) Feasibility- Can the proposed research effectively be done in the proposed population given the parameters of this study design?
5) Biases- Has the PI considered all potential biases, if any, that may affect their methodology and results?

Linked App (sig grant) - do not delete
AIMEPCCo: American Indian Men's Experiences with Prostate Cancer Communication

Which grant program is this application for?
Greater Value Portfolio

PI(s)
Christopher Gillette

Project Title
AIMEPCCo: American Indian Men's Experiences with Prostate Cancer Communication

1) Are the objectives of the research achievable with the proposed research methods?
The objectives of the research are achievable with the proposed research methods.

The objectives of this research are to:
Aim 1: Characterize facilitators and barriers to PC-related communication, explore role preferences in SDM, and identify preference-relevant features that influence PC screening among Lumbee men.
Aim 2: Elicit the PC-related communication SDM role, and screening preferences of Lumbee men and elicit barriers to and facilitators of PC screening uptake using a DCE survey.
Aim 3: Estimate the SDM role and screening preferences among Lumbee men, describe trade-offs, and identify feasible, acceptable, preference-concordant PC communication and screening strategies that are specifically targeted at Lumbee men.

2) Is there a clear description of the dataset that will be used in the analysis, including the purpose for which the data were originally collected, any checks used to assure the validity of the data, and a description of potential non-random biases that might impact the data?
• Providing a description of preliminary data, if available, would strengthen the application. The data will be prospectively collected, but a more thorough description of biases that might impact the data would also strengthen the application.
• The dataset that will be used in the analysis…. will be prospectively collected from interviews with up to 30 men for the DCE formative mixed-methods phase. For the DCE questionnaire, 150 Lumbee men will be recruited from the UNC Health Southeastern health system.
• The original purpose of the collected data was to…. not applicable, the data will be prospectively collected specifically for this study.
• The researchers will assure the validity of the data by…"engage in member-checking with 5 interview participants to ensure that researcher bias did not misinterpret any of the qualitative findings. " It is also mentioned bias will be minimized through regular application of standard validity tests to incoming DCE data.
• The non-random biases that may impact the data are… discussed briefly in the limitations. Only a mention of anticipating "varying interpretations of the guidelines by clinicians based on subjectivity in scoring, quality of care guided by the EOL measure" and that "the proposed recruitment methods implies potential biases from self-selection." However, a number of non-random biases may be present in data (e.g., missing data), but it is not clear how this would be addressed.

3) Are the outcome measures already specified and appropriate to the research aims?
• The intended outcome measures for this research are already specified and appropriate. The outcomes relevant to each aim are appropriate.

4) Are the proposed analytic methods appropriate to the research question and dataset?
• The proposed analytic methods are appropriate to the research question and dataset.
• The proposed analytic methods involve….a mixed methods research approach for Aim 1. Much of this aim is focused on interviewing participants with qualitative methods and is well described. Participants will also complete a quantitative survey and descriptive statistics will be used to summarize it.
• Aim 2 plans to use a fractional factorial design of the questions and Aim 3 involves a statistical analysis of preferences.

5) Do you have any concerns that any of the research questions, protocols, sampling, or data analyses may inadvertently mask or exacerbate racial, ethnic, or gender health inequities? Please explain your answer
• The PI intends to prevent inadvertently masking or exacerbating racial, ethnic, or gender health inequities by…Not applicable, this application focuses on AIAN men (male members of the Lumbee Tribe), so directly addresses a specific subgroup already that experiences help disparities.

• The following components of the proposed research raise concerns…. None noted. A strength of this application is that it is being done in collaboration with the Tribal Council.
• I have no concerns that the research questions, protocols, sampling, or data analyses may inadvertently mask or exacerbate racial, ethnic, or gender health inequities.

Please refer to the statistical review guidelines for clarification on the below statements.

1) Based on my statistical review I find all aspects of the evaluation criteria appropriate for the proposed research
2) Based on my statistical review I find most aspects of the evaluation criteria appropriate for the proposed research with some changes and improvements
3) Based on my statistical review I find fatal flaws in the criteria I evaluated for the proposed research (if you indicate that you find fatal flaws please include them in your additional comments/concerns)

Overall Review Please choose the statement above that best represents your overall review of the research proposal you reviewed.
1

Additional Comments/Concerns
The statistical approach for each aim is well-described and the team has experience using these methods. The study uses both qualitative and quantitative methods. This research will likely be generalizable to the population of Lumbee men, but may not generalize to other AI men due to selection bias. Plans for bias due to missing data (e.g., survey questions not answered) or selection bias would strengthen the application. For example, comparing the characteristics of Lumbee men that agree to participate versus the general population, if possible, might suggest whether certain characteristics are related to participation.

For each statement below please use the following scale: strongly disagree, disagree, agree, or strongly agree, to indicate the degree to which you agree or disagree.

The objectives of the proposed research aligned are achievable with the proposed research methods
Strongly Agree

The research methods are most appropriate for the proposed research
Strongly Agree

This research will produce results that can be generalizable
Disagree

The proposed research effectively be done in the proposed population given the parameters of the chosen study design
Strongly Agree

The PI has considered all potential biases if any, that may affect their methodology and results
Disagree

App Committee Feedback:

Linked Application (do not delete)
AIMEPCCo: American Indian Men's Experiences with Prostate Cancer Communication

Which grant program is this application for?
Greater Value Portfolio

PI(s) Name
Christopher Gillette

Project Title
AIMEPCCo: American Indian Men's Experiences with Prostate Cancer Communication

Please thoroughly write your responses to the questions below. The questions in light gray are for your consideration for the overarching question. Your critique will be shared, anonymously, with the applicants as part of the feedback they receive from the Foundation on their proposal.

1) Overall, how well does the proposed project align with the purpose of the GVP program?
Does it focus on achieving value?
Yes. The development of a culturally-relevant prostate cancer shared decision making intervention to improve screening and treatment among American Indian men aligns with the GVP program.

Does it align with the improvement initiatives and strategic goals of the partnering organization?
The research team is partnering with the Lumbee Tribe of NC and is aligned with the goals of the organization – but I wish the application included a letter of support from the tribe..

Is there scientific merit to the research question being asked?
Yes, there is good evidence that the use of culturally appropriate decision making tools can improve care for American Indians. There is well developed evidence for interventions related to smoking and weight loss, but less information about how a culturally appropriate intervention may help to improve the use of prostate cancer screening.

2) Are the objectives of the research achievable with the proposed research methods?
Yes.

Is there a clear description of the dataset that will be used in the analysis, including the purpose for which the data were originally collected, any checks used to assure the validity of the data, and a description of potential non-random biases that might impact the data? Yes.

Do you have any concerns that any of the research questions, protocols, sampling, or data analyses may inadvertently mask or exacerbate racial, ethnic, or gender health inequities? My only concern relates to the willingness of Lumbee Tribe members to participate. The fact that Dr. Bell is both a Co-I and a member of the tribe helps to reassure me that the team will have credibility and will be seen as trustworthy by the tribe.

Are the outcome measures already specified and appropriate to the research aims? Yes.

Are the proposed analytic methods appropriate to the research question and dataset? Yes.

3) Does the partnership between the research team and the partnering organization appear it will be effective in achieving the goals of the study?
Are the key personnel from the partnering organization the appropriate ones to be working with this study?
As I indicate above, the inclusion of Dr. Bell on the team is helpful. The proposal makes reference to the use of community based participatory research but it would be useful if the proposal included a bit more detail about this.

Has the applicant successfully made the case that the partnership is integral to achieving the aims of the study? Yes.

From the perspective of the partnering organization, how will the proposed research assist in achieving their goals as opposed to a more traditional quality improvement approach?
Yes, the use of CBPR goes beyond most traditional QI improvement projects.

Do the objectives and timeline for the study line up well with the partnering organization's improvement or adoption goals? Yes

4) Are the knowledge transfer and integration or implementation activities described appropriate and attainable?

Are the approaches described likely to facilitate the implementation of positive results that may arise from the study? Yes

Are the practices and policies of the researcher’s institution and the partnering organization concerning data sharing, publishing, and other dissemination activities clear and appropriate? Yes

5) Are the resources for conducting the study adequate and appropriate for completing the study?
Are the types of personnel and their time commitments appropriate for this study? Yes, but some of the Co-Is are covered at a relatively low level.

Does the budget properly account for needed expenditures? Yes

Is the overall cost suitable given the project goals and activities? Yes

Additional Comments/Concerns

App Committee Feedback:

Linked Application (do not delete)
AIMEPCCo: American Indian Men's Experiences with Prostate Cancer Communication

Which grant program is this application for?
Greater Value Portfolio

PI(s) Name
Christopher Gillette

Project Title
AIMEPCCo: American Indian Men's Experiences with Prostate Cancer Communication

Please thoroughly write your responses to the questions below. The questions in light gray are for your consideration for the overarching question. Your critique will be shared, anonymously, with the applicants as part of the feedback they receive from the Foundation on their proposal.

1) Overall, how well does the proposed project align with the purpose of the GVP program?
Yes.
• It focuses on achieving value for a well defined, historically disadvantaged population.
• It aligns well with the improvement initiatives and strategic goals of the partnering organization.
• The research question being asked has scientific merit.

2) Are the objectives of the research achievable with the proposed research methods?
Yes. Researchers will use the discrete choice experiment (DCE) methodology commonly used to elicit patients' preferences. The DCE model is grounded in the economic theory of utility maximization The project involves a sequence or surveys and interviews of 40-69 y.o. Lumbee Indian males to identify and quantify barriers, facilitators, ordinal preferences from a set of alternatives and acceptable trade-offs. These steps are intended to "predict" the most likely structure of a SDM to promote higher rates of PC screening. The researchers consider this method superior to other approaches of iterative trial-and-error implementations and post-hoc adaptations of failed programs.
Aim 1: Characterize facilitators and barriers to PC-related communication, explore role preferences in SDM, and identify preference-relevant features that influence PC screening.
Aim 2: To elicit the PC-related communication SDM role, and screening preferences of Lumbee men and elicit barriers to and facilitators of PC screening uptake using a DCE survey.
Aim 3: Identify feasible, acceptable, preference-concordant PC communication and screening strategies that are specifically targeted at Lumbee men. There is no explicit goal of making the SDM generalizable to other populations although they state that the research may "hold promise to improve AI men's PC outcomes".
• I do not have any concerns that any of the research questions, protocols, sampling, or data analyses may inadvertently mask or exacerbate racial, ethnic, or gender health inequities. There is no explicit goal of making the SDM generalizable to other populations therefore, the "selection bias" of designing a SDM for Lumbee Indian males cannot be considered a weakness.
• The outcome measures already specified and appropriate to the research aims.
• The proposed analytic methods are appropriate to the research question and dataset. The methods are clearly described. In addition to surveys and interviews researchers will use "talking circles", which in the Lumbee community is a culturally-relevant method of sharing information, showing support, and solving problems. The data will be collected prospectively to quantify the relative importance placed on one feature level in comparison with other feature levels evaluated in the DCE. The statistical analysis is appropriate for this study design.

3) Does the partnership between the research team and the partnering organization appear it will be effective in achieving the goals of the study?
Yes. The study design is well aligned with the partnering organization goal of improving PC screening among Lumbee Indian males, age 40-69. The study will be being conducted with a research team across three academic research institutions: Wake Forest University School of Medicine and the University of North Carolina at Chapel Hill in North Carolina and the University of South Carolina. The team has expertise in mixed-methods research, cancer screening, American Indian research and DCE development and implementation. Dr. Ronny Bell is an enrolled member of the Lumbee tribe and has extensive experience in conducting research in American Indian tribes and the Lumbee tribe in particular. Researchers have met with Dr. Joseph Roberts, an enrolled member of the Lumbee tribe and Medical Director of UNC Southeastern Health, who has expressed his support for our study.
• From the perspective of the partnering organization, the proposed research will assist in achieving their goals using the DCE model grounded in the economic theory of utility maximization, as opposed to other similar traditional quality improvement approaches.

• The objectives and timeline for the study line up well with the partnering organization's improvement or adoption goals.

4) Are the knowledge transfer and integration or implementation activities described appropriate and attainable?

1. Yes

• The approaches described are likely to facilitate the implementation of positive results that may arise from the study.

• The practices and policies of the researcher's institution and the partnering organization concerning data sharing, publishing, and other dissemination activities clear and appropriate. Results will be present to the. Lumbee Tribe through the monthly Tribal Council meetings and to the to the leadership of UNC Southeastern Health, organizations that are partnering with the research team to identify Lumbee men.

5) Are the resources for conducting the study adequate and appropriate for completing the study?

1. Yes

• The types of personnel and their time commitments are appropriate for this study.

• The budget properly account for needed expenditures.

• The overall cost is suitable given the project goals and activities.

Additional Comments/Concerns

First-of-a-kind proposals focused on a well defined population is attractive because it can lay solid foundations for subsequent, large scale research. To my knowledge, this is the first GVP proposal using the DCE model. This methodology seems highly efficient for the purpose of designing a SDM tool where patients must rank realistic multiple choices and accept trade-offs based on their values and preferences. This proposal introduces a novel (at least to this reviewer) method for researchers investigating a wide range of topics, from patient preferences to complex policy decisions.

App Committee Feedback:

Linked Application (do not delete)
AIMEPCCo: American Indian Men's Experiences with Prostate Cancer Communication

Which grant program is this application for?
Greater Value Portfolio

PI(s) Name
Gillette

Project Title
AIMEPCCo: American Indian Men's Experiences with Prostate Cancer Communication

Please thoroughly write your responses to the questions below. The questions in light gray are for your consideration for the overarching question. Your critique will be shared, anonymously, with the applicants as part of the feedback they receive from the Foundation on their proposal.

1) Overall, how well does the proposed project align with the purpose of the GVP program?
Strengths
• Prostate cancer screening is a preference sensitive screening; although screening has benefits in identifying cancer, there are also costs to the individual (e.g., false positives) and using shared decision making approaches to communication has the potential to improve decision making

Weaknesses
• The plan for developing the 2-3 question screening survey/decision aid on preferences is out of scope of the proposed research, and validating the survey will require additional time and resources before it could be implemented; additionally, it is not clear where the provider communication strategies themselves will be developed

2) Are the objectives of the research achievable with the proposed research methods?
Strengths
• The collection of attributes in Aim 1 and ranking of those attributes in Aim 2 are reasonable for developing potential communication and screening strategies targeted at Lumbee men

Weaknesses
• Although the proposal makes the case that they can recruit sufficient numbers of men for the qualitative and quantitative phases, recruitment relies heavily on men being seen at UNC Southeastern Health, and many men may not be seen annually, restricting recruitment. Data on the number of eligible men by recruitment strategy would be helpful to demonstrate feasibility.
• Aims 2 and 3 are largely overlapping, with Aim 2 conducting the DEC survey and Aim 3 analyzing the data and identifying strategies

3) Does the partnership between the research team and the partnering organization appear it will be effective in achieving the goals of the study?
Strengths
• There is a strong partnership with the Lumbee Tribe of North Carolina, including co-investigators who are members of the Lumbee Tribe and existing approval from the tribal council for the project.
• There are clear methods for which the partnering organizations will provide input that are culturally aligned (e.g., talking circles, tribal council)

Weaknesses
• UNC Southeastern Health appears to be a partner only as a recruitment site and is a relatively modest partnership
• The budget for the partnering organizations is relatively small (5% for Bell, 2.5% for Reuland)

4) Are the knowledge transfer and integration or implementation activities described appropriate and attainable?
Strengths
• There is a plan to disseminate findings through the Lumbee Tribal Council via monthly meetings at 3 time points

Weaknesses
• The plan for how these results will lead to future research in the design of pragmatic trials is underdeveloped
• The plan for developing the 2-3 question screening survey on preferences is out of scope of the proposed research

5) Are the resources for conducting the study adequate and appropriate for completing the study?
Strengths
• There are stipends for participant engagement in the interviews and DCE

Weaknesses
• A substantial portion of the budget is going to consultant costs – DEC expert ($10200 in Year 1) and a consultant to deliver the DCE and lead talking circles and for a DCE expert ($37140 in Year 2, even though Dr. Gillette has DCE expertise and will be covered at 30%.
• Roles of some investigators are underdeveloped. More detail on who will be doing the work related to the research itself would be helpful, given the large role of consultants and the QPRO shared resource.

Additional Comments/Concerns
• The proposal is lacking data/case about whether AIAN men have lower rates of screening / higher rates of prostate cancer compared to other men. The significance of the project is lacking some data on magnitude of the problem and relies heavily on speculation – e.g., AIAN men may not perceive the current ways of screening as being respectful, rates of screening may be lower. The proposed line of research may benefit from some additional preliminary research to justify the project significance. Although the NAMCS paper provides data on PC screening in AIAN men, it is not clear that NAMCS is representative at this level of data. It is likely that data are not available for many areas to support the need, but more detail is needed regarding where data are lacking and what is speculation.
• Overall, this project has a number of strengths, including a strong partnership with community organizations, and individuals who are members of the Lumbee Tribe. The proposed project represents a creative use of DCE including a first stage of research that elicits attributes of communication regarding prostate cancer screening, and the second stage of research that utilizes a DCE to elicit ranking of preferences. There are strategies to obtain feedback and share findings with the community. Limitations relate to underdevelopment of the significance of the research, a screening survey being out of scope of the proposed research, and lack of details regarding the budget allocation.

11/01/23

Christopher Gillette, PhD
Wake Forest University Health Sciences
Medical Center Blvd.
Winston-Salem, NC 27157

RE: AIMEPCCo: American Indian Men's Experiences with Prostate Cancer Communication

Dear Dr. Christopher Gillette,

The Trustees of The Patrick and Catherine Weldon Donaghue Medical Research Foundation are pleased to advise you that a **$426,476** grant has been awarded to your host institution, **Wake Forest University**, for support of your research as described in the application submitted to the ***Greater Value Portfolio*** grant program.

The mission of the Foundation is to be an imaginative, collaborative, and engaged participant in the process that begins with rigorous health research and ends with realized health benefits. As a way of fulfilling this mission, two of the Foundation's goals are 1) to provide support for rigorous research that more directly leads to a positive impact on health and 2) to contribute to efforts that optimize the capacity of health research to address the needs of policymakers, practitioners, and consumers. The Foundation developed the ***Greater Value Portfolio*** grant program to advance its mission and these goals.

This ***Greater Value Portfolio*** grant award is made specifically for you to pursue the activities that you described in your application. Payment on this grant will be made to your host institution, pending the return of this five-page agreement. The grant period will begin on **01/01/2024**, and end on **12/31/2025**.

It is your obligation and your host institution's obligation to notify the Donaghue Foundation of any change that will substantially alter the circumstances and commitments described in the original application. Notification should occur as soon as practicable and, under normal circumstances, at least 30 days prior to such change. If you change your affiliation to another institution your award will lapse if we have not approved its continuation and the unexpended funds will revert to the Donaghue Foundation.

Please note that the Donaghue Foundation has established policies regarding changes in declared budgets, nepotism, retention of rights to intellectual property (patents, etc.), and uses of interest earned, as well as other matters. By signing this agreement, you and your host institution, indicate acceptance of this grant and of Foundation funds and agree to be subject to these policies. The policies are available on our website at www.donaghue.org. Please contact Shamira Chappell, Program Manager, at chappell@donaghue.org if you have any questions.

The Donaghue Foundation

Please sign at the end of this five-page award notice, obtain the appropriate institutional signature, and return it to this office to indicate your acceptance and your host institution's acceptance of this grant and agreement to the stated terms and conditions. The Foundation will provide a copy of the fully executed agreement via email once it becomes available.

We would like to extend our congratulations to you for the excellence you have already demonstrated and our best wishes in your research endeavors.

Sincerely,

Stacy Cloud X_______________________________ Date:
President, Donaghue Foundation

Lynne Garner, PhD X_______________________________ Date:
Trustee, Donaghue Foundation

Amy R. Lynch X________________________________ Date:
Senior Vice President
Bank of America, N.A.
Trustee, Donaghue Foundation

Enclosure: Attachment 1: Conditions of the Grant

Attachment 1: Conditions of Grant

Payment Schedule
Year 1-$**220,719** by **01/15/2024**
Year 2-$**205,757** to be paid by **01/15/2025** pending acceptance of Year 1 reports

Reporting Schedule
Narrative and Financial progress reports
Interim Report-Due by **10/01/2024** (9-month report)
Final Report-Due by **03/02/2026**

Narrative reporting requirements will be provided by the Foundation. Financial reports must be completed by your grants & contracts office or finance department. The final narrative & financial reports are due 90 days after the grant term ends.

Grantee Call Schedule

The Foundation requires participation in grantee check-in calls. The Foundation will provide ample notice to secure the exact date and times for the calls and will be mindful of the grantee's schedule.

Call date(s):
TBD

Use of Award
As you may know, the laws of the United States place certain restrictions on the manner in which funds awarded to organizations by charitable trusts and foundations may be expended. For example, although we hope that research results will affect health policy and practice through educational activity, Foundation funds may not be used for the specific purposes of engaging in political campaigns or efforts to influence the outcome of specific legislation.

The grant monies which you receive, including any interest earned thereon, may be used only for the purpose(s) stated in this agreement. Funds may not be expended for any other purpose without the prior written approval of The Patrick and Catherine Weldon Donaghue Medical Research Foundation.

In order for us to carry out our legal responsibilities, we require that the grantee abides by the following requirements:

Reporting Requirements
The grantee must furnish a final written report to the Donaghue Foundation in accordance with the requirements set out in this agreement. This report must contain a narrative account of what was accomplished by the expenditure of funds (including a description of progress toward achieving the goals of the project supported by the grant), and a detailed financial accounting of expenditures made under the terms of the grant. If consortium agreements or sub-contract arrangements are included in grant budgets, appropriate reporting must be filed by consortium or subcontracting organizations.

Attribution
In any publication of work done under this award, acknowledgment must be given to "The Patrick and Catherine Weldon Donaghue Medical Research Foundation." This is because the Foundation's creator, Ethel Donaghue, intended that work funded by the Foundation would serve in part as a memorial to her parents, Patrick Donaghue and Catherine Weldon Donaghue.

Evaluation of the Grant
At its own expense, The Donaghue Foundation may monitor and conduct an evaluation of operations under this grant. The evaluation may include visits to the grantee by representatives of the Donaghue Foundation to observe and discuss the funded program.

Grantee's Financial Responsibilities
The grantee must keep systematic records of all expenditures relating to this grant. Vouchers, consisting of bills, invoices, canceled checks, receipts, etc., must be retained by the grantee for at least five (5) years after the close of the grant period and must be available for inspection by representatives of the Donaghue Foundation at reasonable times during this period. The Donaghue Foundation may, at its own expense, examine, audit, or cause to be audited the records of the grantee insofar as they relate to activities supported by this grant.

Unused Funds and Reversion
Because the grant is intended to support a particular project and to provide support for a specific period, any portion of the grant not expended at the completion of the project or grant period must be returned to the Donaghue Foundation within 90 days thereof.

If any of the following events occur, the grantee agrees to promptly give written notice to the Donaghue Foundation and to repay upon demand all portions of the grant which are within the grantee's control; moreover, the Donaghue Foundation may withhold or cancel all unpaid installments of the grant:

- The grantee ceases to be exempt from Federal Income Taxes under Section 501c(3) of the Internal Revenue Code, or becomes a private foundation under Section 509(a) of the Code.
- There is a material change in the purpose, character, or method of operation of the grantee such as to jeopardize its tax-exempt status.
- A determination, preliminary or otherwise, is made by the Internal Revenue Service that the grant does not constitute a qualifying distribution or is a taxable expenditure by the Donaghue Foundation, within the meaning of the Internal Revenue Code.
- The grantee fails, in the judgment of the Donaghue Foundation, to perform one or more of its duties as required by the terms of this letter.

Non-Compliance
In the event of material noncompliance with this Agreement (including the misappropriation of any Grant funds), the Foundation shall have the right to 1) suspend or discontinue its support of the project, including termination of this Agreement; 2) require that the grantee institution discontinue the use of the Foundation's name or the Donaghue family name in connection with the project; 3) withhold payment of any Foundation funds reserved for the project, either temporarily or permanently; and/or 4) demand repayment of unexpended grant funds from the project previously paid under this Agreement, with the exception of allowable costs and obligations that cannot be canceled and were properly incurred prior to the notice of termination

In making this grant, The Patrick and Catherine Weldon Donaghue Medical Research Foundation assumes no obligation to provide other or additional support to the grantee. Kindly confirm your acceptance of this award and all the conditions stated or referred to above, by signing and returning the enclosed copy of this five-page award notice.

We acknowledge that we have received and retained a copy of this agreement. The terms and conditions described above are acceptable and we agree to comply with them and to be bound by the established policies of the Donaghue Foundation, to which the grant is subject.

____

Signature – Institutional Officer Date

Title____

Print Name____

Organization____

____

Signature – Principal Investigator Date

Title____

Print Name____

Organization____
